# A chromosome-level genome assembly of a deep-sea starfish (*Zoroaster* cf. *ophiactis*)

**DOI:** 10.1038/s41597-023-02397-4

**Published:** 2023-08-01

**Authors:** Jun Liu, Yang Zhou, Yujin Pu, Haibin Zhang

**Affiliations:** 1 Institute of Deep-Sea Science and Engineering, Chinese Academy of Sciences, Sanya, Hainan China; 2 University of Chinese Academy of Sciences, Beijing, China

**Keywords:** Comparative genomics, Genome

## Abstract

Understanding of adaptation and evolution of organisms in the deep sea requires more genomic resources. *Zoroaster* cf. *ophiactis* is a sea star in the family Zoroasteridae occurring exclusively in the deep sea. In this study, a chromosome-level genome assembly for *Z*. cf. *ophiactis* was generated by combining Nanopore long-read, Illumina short-read, and Hi-C sequencing data. The final assembly was 1,002.0 Mb in length, with a contig N50 of 376 Kb and a scaffold N50 of 40.4 Mb, and included 22 pseudo-chromosomes, covering 92.3% of the assembly. Completeness analysis evaluated with BUSCO revealed that 95.91% of the metazoan conserved genes were complete. Additionally, 39,426 protein-coding genes were annotated for this assembly. This chromosome-level genome assembly represents the first high-quality genome for the deep-sea Asteroidea, and will provide a valuable resource for future studies on evolution and adaptation of deep-sea echinoderms.

## Background & Summary

Sea stars or starfish, members of the class Asteroidea, are one of the five extant groups within Echinodermata. Asteroids are a diverse group including about 1,900 extant species^1^. Asteroids occur worldwide in various marine habitats from the intertidal to the hadal zone (~10,000 m)^2^. As major predators, asteroids play important roles in marine ecosystems by affecting the ecology of the prey and the community structure^3^. With a long fossil record, sea stars are of tremendous interest of paleontologists and evolutionary biologists^4^. The remarkable life history diversity in the Asteroidea make them good subjects for studies of evolutionary developmental biology, developmental ecology and regeneration^5–7^.

Of all the extant starfish families, approximately half occur exclusively in the deep sea (>200 m), and many families among others also comprise deep-sea members^1^, suggesting a high diverse of asteroids in the deep-sea floor. Sea stars of the family Zoroasteridae (order Forcipulatida), occurring exclusively in the deep sea (~200–6,000 m), are prominent members of the deep-sea benthic animals, and they are often collected in high densities, suggesting their potentially important roles in the deep-sea ecosystems^8^. Zoroasteridae includes seven genera and approximately 40 species, and is phylogenetically basal among Forcipulatida^1^, implying an important evolutionary role of this asteroid group.

It is well known that the deep sea is a unique environment that is mostly characterized by darkness, low temperatures, high hydrostatic pressure and limited food resources^9^. The harsh environment in the deep sea challenges organisms living there. Recently, several deep-sea animal species, such as sea cucumber^10^, marine mussel^11^, limpet^12^, cold-water coral^13^, anemone^14^, tubeworms^15–17^ and fish^18,19^, have been investigated through the genomic data, demonstrating molecular mechanisms of adaptation to the deep sea. As one of the main members of the sea floor, however, genomic resources for the diverse starfish at the chromosome level are scarce^20–25^, and no genomic resources has been available up to now for the deep-sea starfish, which hinders studies on their evolution, speciation and adaptation to the deep sea.

In the present study, we present a chromosome-level genome assembly for the deep-sea starfish, *Zoroaster* cf. *ophiactis*, the first high-quality genome assembly for the deep-sea Asteroidea. The species, belonging to the deep-sea asteroid family Zoroasteridae, was collected at depth of 1,750 m in the South China Sea. A combined strategy involving Nanopore long-read, Illumina short-read and Hi-C sequencing technologies was used in this study. This high-quality genome will serve as a valuable resource for future studies on the adaption and evolution of deep-sea starfish.

## Methods

### Sample collection

One specimen of the starfish *Z*. cf. *ophiactis* was collected in the northern South China Sea (111.033E, 17.597 N, 1750 m in depth) by the manned submersible Shenhai Yonghshi during the cruise TS07 of R/V Tansuo 1 in 2018. Tissues of one arm were frozen with liquid nitrogen and then kept at −80 °C until further use.

### DNA extraction, library preparation and sequencing

High molecular weight (HMW) genomic DNA was extracted from the frozen tissues by using the SDS method and then purified with the QIAGEN® Genomic kit (QIAGEN) following the manufacturer’s instructions. The quality of the extracted DNA was assessed using 1% agarose gel electrophoresis, and NanoDrop™ One UV-Vis spectrophotometer (Thermo Fisher Scientific, USA) with the OD 260/280 of 1.8–2.0 and OD 260/230 of 2.0–2.2. The quantity of the DNA was measured by Qubit® 3.0 Fluorometer (Invitrogen, USA). DNA libraries for Illumina sequencing were prepared using the Truseq Nano DNA HT Sample Preparation Kit (Illumina USA) according to the manufacturer’s protocols. The libraries were sequenced on the Illumina Hiseq 4000 platform, yielding 150-bp paired-end reads with an insert size of ~350 bp. In total, ~103 Gb of Illumina raw reads were obtained. For the Oxford Nanopore library preparation, genomic DNA fragments > 20 kb were selected using the BluePippin system (Sage Science, USA). Approximate 2 µg HMW DNA was used as input material, according to the manufacturer’s instructions, for the ligation Sequencing kit SQK-LSK109 (Oxford Nanopore Technologies, UK). Sequencing was performed on a Nanopore PromethION sequencer (Oxford Nanopore Technologies, UK). A total of ~60 Gb of Nanopore raw reads were generated. A high-throughput chromatin conformation capture (Hi-C) method was applied to generate a chromosome-level genome. Briefly, the frozen arm tissues were crosslinked with 2% formaldehyde, and then digested with the restriction enzyme MboI (400 units). The DNA ends were tagged with the biotin-14-dCTP and fragments were sheared to 200–600 bp. The resulting Hi-C library was sequenced on the Illumina HiSeq 4000 platform (paired-end 150 bp reads). A final ~72 Gb of raw reads were obtained.

### RNA extraction and transcriptome sequencing

The total RNA was isolated from the frozen arm tissue using Trizol (Invitrogen, Carlsbad, CA, USA), following the manufacturer’s instructions. Concentration of the isolated RNA was measured using the NanoDrop 2000 spectrophotometer (Thermo Fisher Scientific, USA), and its quality was evaluated by 1.5% agarose gel electrophoresis. RNA integrity was quantified by the Agilent 5400 fragment analyzer (Agilent, USA). RNA-seq libraries were constructed by the NEBNext® Ultra™ RNA Library Prep Kit (NEB, USA) following the manufacturer’s instructions. Libraries were then sequenced on an Illumina Hiseq 4000 platform (paired-end 150 bp reads). A total of ~8 Gb raw reads were yielded and used for the gene prediction.

### Genome assembly

**Fig. 1:**
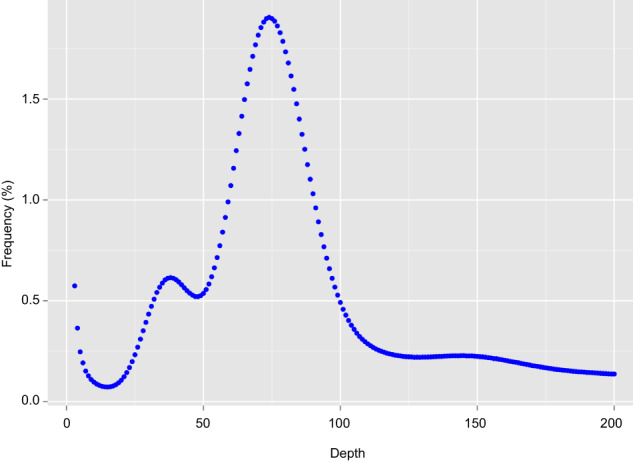
K-mer distribution (K = 19) of *Zoroaster* cf. *ophiacti* genome. The x-axis is the k-mer depth, and the y-axis represents the corresponding frequency of the k-mer at a given depth.

Genome size, proportion of repetitive sequences and heterozygosity was estimated by using the Illumina short-read data and the k-mer analysis with Jellyfish v2.3.0^26^. Based on the ~103 Gb Illumina data and the 19-mer frequency distribution analysis, a total of 78,106,733,386 k-mers were obtained after removing k-mers with abnormal depth, and the 19-mer peak was at a depth of 74. Therefore, the genome size of *Z*. cf. *ophiactis* was estimated to be 78,106,733,386/74 = 1,055 Mb, the heterozygosity was about 0.32%, and the proportion of repetitive sequences was roughly 69.85% (Fig. 1).

**Fig. 2:**
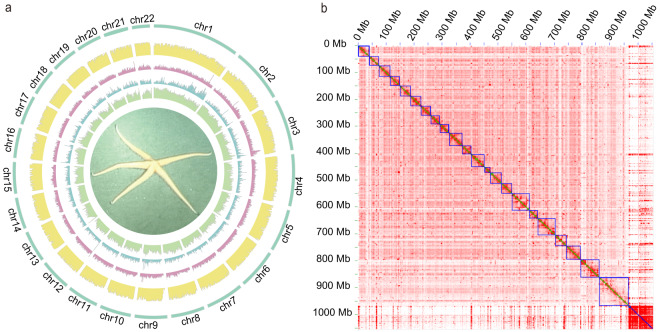
Characteristics of the genome assembly of *Zoroaster* cf. *ophiactis*. (**a**) Genome overview of the 22 chromosomes. Tracks from inner to outer represent repeats coverage (19–96%), genes density (1–85), GC content (35–47%), genome sequence depth (7–100 X), and assembled chromosomes, respectively, with densities calculated within a 500-kb window. (**b**) Hi-C contact map produced by 3D-DNA. The blue square represents a pseudo-chromosome, and small green squares inside each blue square are contigs that make up the chromosome.

The Nanopore long-read data were used to generate a contig-level assembly for the *Z*. cf. *ophiactis* genome. A preliminary assembly was generated by using the program WTDBG2 v2.5^27^ (parameters: -p 19 -AS 2 -s 0.05 -L 5000 -t 36 -fo starfish). Then, three rounds of polishing were carried out with ~103 Gb of Illumina reads by the software Nextpolish v1.2.0^28^. The Hi-C technology was used for chromosome-level genome assembly. Raw Hi-C paired reads were trimmed by Fastp v0.20.0^29^, and aligned to the draft assembly with Juicer v1.5.7^30^ using default settings. Contigs were scaffolded using 3D-DNA pipeline v180114^31^ with all valid Hi-C reads. The chromosome-scale scaffolds were adjusted manually using Juicebox v1.11.0812^32^ with the aid of the Hi-C contact map whereby redundant contigs and misjoins were removed and fixed. All the corrections were incorporated into the assembly using the 3D-DNA post-review pipeline. Ultimately, the contigs were anchored to 22 pseudo-chromosomes, accounting to 92.3% of the total genome (Table 1; Fig. 2). The lengths of the 22 pseudo-chromosomes ranged from 22.2 Mb to 107.2 Mb (Table 2). The final assembly was 1,002.0 Mb in length, containing 8,895 congtigs with N50 of 376 kb and 616 scaffolds with scaffold N50 of 40.4 Mb (Table 1).

**Table 1:** Summary statistics of *Zoroaster* cf. *ophiactis* genome assembly.

Feature	Value
Assembly genome length (Mb)	1,002.0
Repeat region in genome (Mb)	673.3
Contigs number	8,895
Contigs N50 (kb)	376.2
Contigs N90 (kb)	36.8
Longest Contig (kb)	898.5
Scaffolds number	616
Scaffolds N50 (Mb)	40.4
Scaffolds N90 (Mb)	30.9
Longest scaffold (Mb)	107.2
Number of chromosomes	22
Chromosome/total	92.3%

**Table 2:** Pseudo-chromosome length statistics after Hi-C assisted assembly.

Pseudomolecule	Length (bp)
chr_1	107,181,989
chr_2	65,707,080
chr_3	61,886,573
chr_4	61,736,829
chr_5	50,998,033
chr_6	48,773,539
chr_7	46,547,494
chr_8	41,726,941
chr_9	40,360,388
chr_10	38,901,745
chr_11	38,538,917
chr_12	37,951,288
chr_13	37,900,789
chr_14	37,866,290
chr_15	37,463,515
chr_16	35,801,734
chr_17	35,469,185
chr_18	32,573,505
chr_19	31,984,754
chr_20	30,912,062
chr_21	30,806,620
chr_22	22,172,998

### Repeat annotation

Repetitive elements in the genome assembly were annotated by using RepeatModeler v2.0.1^33^, RepeatMasker v4.0.7^34^ and TRF v4.0.9^35^. Ultimately, a total of 673.3 Mb repeat sequences were identified, accounting for 63.9% of the whole genome. The DNA elements (114.3 Mb) were the predominant type of the transposable elements (TEs), which represented 10.84% of the genome, followed by the long interspersed nuclear elements (LINEs) with the portion of 7.69% in the genome. The short interspersed nuclear elements (SINEs) and the long terminal repeat (LTR) retrotransposons occupied 2.86% and 2.95% of the genome, respectively.

### Gene prediction and annotation

Protein-coding genes were predicted with three different strategies: *ab initio* gene prediction, homology-based prediction, and transcript prediction. The *ab initio* gene prediction was performed using Augustus v2.4^36^, and GlimmerHMM v3.0.4^37^. For the homology-based prediction, protein sequences from five echinoderm species, *Acanthaster planci* (GCF_001949145.1)^38^, *Anneissia japonica* (GCF_011630105.1)^39^, *Apostichopus japonicus* (GCA_002754855.1)^40^, *Lytechinus variegatus* (GCF_018143015.1)^41^ and *Strongylocentrotus purpuratus* (GCF_000002235.5)^42^, were downloaded from the NCBI database for the gene prediction as implemented in TblastN v2.2.29^43^ with an e-value ≤ 1e-5. For the transcriptome-based annotation, clean RNA-seq reads were aligned to the *Z*. cf. *ophiactis* genome assembly by using HISAT2 v2.2.1^44^, and gene set was predicted by using PASA v2.3.2^45^ pipeline. Finally, results from *ab initio* prediction, homology-based prediction, and transcript prediction were integrated by using EvidenceModeler v1.1.1^46^ to generate a consensus and non-redundant gene set. Overall, 39,426 protein-coding genes were annotated for the *Z*. cf. *ophiactis* genome by combining three different methods, with an average of exon and intron length of 217.7 bp and 1952.8 bp, respectively (Table 3). The average length and number of the genes, exons, and introns of the *Z*. cf. *ophiactis* genome were comparable to those reported in other sea stars^24^.

**Table 3:** Statistics of genome annotation.

Feature	Value
Number of predicted protein-coding genes	39,426
Average protein-coding gene length (bp)	4,051.23
Number of exons	214,384
Average exon length (bp)	217.72
Number of introns	174,958
Average intron length (bp)	1,952.84

Functional annotation for the predicted protein-coding genes was performed against six public databases, Kyoto Encyclopedia of Genes and Genomes (KEGG), Gene Ontology (GO), NCBI-NR (non-redundant protein database), Swiss-Prot, SMART and InterProScan with BLASTP v2.2.23^47^ and an e-value cutoff of 1e-5. The results showed that 36,557 (92.72%) predicted genes were annotated by at least one public database (Table 4).

**Table 4:** Function annotation of predicted protein-coding genes.

	Numbers	Percent of all genes (%)
Total genes	39,426	—
BLAST nr	29,312	74.35
Swiss-Prot	14,989	38.02
KEGG	9,922	25.17
SMART	8,870	22.50
InterProScan	21,433	54.36
GO	13,130	33.30
Total annotated	36,557	92.72

## Data Records

All the raw sequencing data of Illumina, Nanopore, and Hi-C obtained in this study have been deposited in the NCBI Sequence Read Archive (SRA) database with the accession numbers SRR22953576- SRR22953579, and SRR24759671 under the BioProject PRJNA891479^48^. The final genome assembly has been deposited in the Science Data Bank of Chinese Academy of Sciences^49^ and the GenBank database under the accession number JAQQFT010000000^50^. Files of genome annotation, repeat annotation, gene functional annotation and gene family expansion have been submitted to the Figshare database^51^.

## Technical Validation

### Assessment of genome assembly

The genome size of *Z*. cf. *ophiactis* was estimated to be about 1,055 Mb based on the 19-mer frequency distribution analysis. The estimation of genome length was consistent with our final genome assembly (1,002 Mb, Table 1). It is noted that the *Z*. cf. *ophiactis* genome assembly is much larger than genomes reported for other asteroids, including species in the order Forcipulatida (402–561 Mb)^21–24^, and those in the other order, Valvatida (384–608 Mb)^20,25,52^. In addition, 22 pseudo-chromosomes were generated for the *Z*. cf. *ophiactis* genome assembly. The chromosome number is consistent with previous karyotyping studies on some asteroids, including species from Forcipulatida^53^. This is also proved by recent genome studies on several starfish species where 22 pseudo-chromosomes were identified by the Hi-C method^22–24^.

To assess the accuracy of *Z*. cf. *ophiactis* genome assembly, the completeness of the genome assembly was assessed using the conserved metazoan gene set “metazoan_odb10” from the Benchmarking Universal Single-Copy Orthologs (BUSCO) v4.0^54^. The genome assembly was found to have a high level of completeness (95.91%). Of the 954 single-copy orthologs, 95.28% were complete and single-copy, 0.63% complete and duplicated, 0.84% fragmented, and 3.25% were missing (Table 5). In addition, clean Illumina short reads used for the genome survey were aligned back to the *Z*. cf. *ophiactis* genome assembly with Burrows-Wheeler aligner (BWA) v0.7.17-r1198^55^. As a result, 99.35% of the short reads were mapped to the genome. Together, these results indicate the high quality of the *Z*. cf. *ophiactis* genome assembly.

**Table 5:** BUSCO analysis of the genome assembly and genes.

Statistic	Genome		Genes	
	Number	Percent (%)	Number	Percent (%)
Complete BUSCOs (C)	915	95.91	926	97.07
Complete and single-copy (S)	909	95.28	918	96.23
Complete and duplicated (D)	6	0.63	8	0.84
Fragmented (F)	8	0.84	7	0.73
Missing (M)	31	3.25	21	2.20
Total	954	—	954	—

### Chromosome synteny

**Fig. 3:**
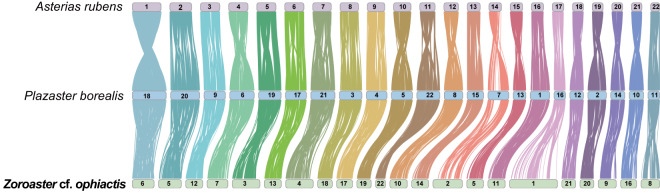
Chromosomal synteny among genomes of *Zoroaster* cf. *ophiactis* and the other two starfish (*Asterias rubens*, and *Plazaster borealis*) in the order Forcipulatida. Numbers in the rectangles represent chromosomes of each genome.

Syntenic relationships among the genomes of *Z*. cf. *ophiactis* and the other two Forcipulatida star fish, *Asterias rubens* (GCF_902459465.1)^56^ and *Plazaster borealis* (GCA_021014325.1)^24^ were inferred and visualization by Blastp and NGenomeSyn v1.37^57^. The three starfish appeared to have very conserved syntenic relationships as every chromosome matched each other well (Fig. 3). This finding provides new evidence of a high level of synteny conservation in the order Forcipulatida^24^.

### Gene annotation validation

To evaluate the completeness of the annotated gene set, we performed the BUSCO analysis using the conserved metazoan database “metazoan_odb10”. The results revealed that 97.07% of the conserved single copy ortholog genes to be complete (96.23% single-copied genes and 0.84% duplicated genes), 0.73% fragmented and 2.2% missing (Table 5). Additionally, functional annotation of the predicted genes revealed that 92.72% of them were annotated by at least one public database (Table 4).

**Fig. 4:**
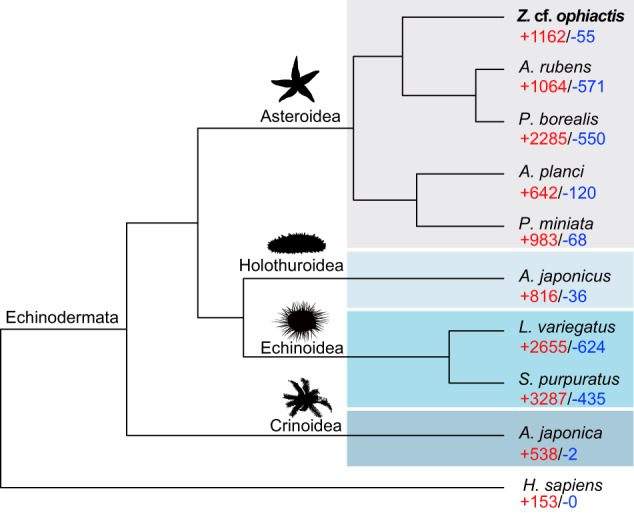
Maximum likelihood phylogenetic tree of *Zoroaster* cf. *ophiactis* and other eight echinoderms. Bootstrap support values for all the nodes are equal to 100. Numbers of expanded (red) and contracted (blue) gene families are shown.

Phylogenetic relationships among *Z*. cf. *ophiactis* and other eight echinoderm species, including *Asterias rubens* (GCF_902459465.1)^56^, *Plazaster borealis* (GCA_021014325.1)^24^, *Acanthaster planci* (GCF_001949145.1)^38^, *Patiria miniata* (GCF_015706575.1)^58^, *Apostichopus japonicus* (GCA_002754855.1)^40^, *Strongylocentrotus purpuratus* (GCF_000002235.5)^42^*, Lytechinus variegatus* (GCF_018143015.1)^41^*, Anneissia japonica* (GCF_011630105.1)^39^, were inferred by using the maximum likelihood (ML) method. *Homo sapiens* (GCF_000001405.39)^59^ was used as the outgroup. Single-copy orthologs among genomes of all species were determined using OrthoFinder v2.3.3^60^ with the default parameters. Multiple alignments of the protein sequences were performed with Muscle v3.8.1551^61^. RAxML v8.2.12^62^ was used to produce the ML trees with the following parameters: -m GTRGAMMA -x 12345 -N 100. The phylogenetic tree was reconstructed with 1,316 single-copy orthologs (Fig. 4). *Zoroaster* cf. *ophiactis* was clustered with *A. rubens* and *P. borealis* within the family Asteriidae, where they all belong to the order Forcipulatida, and then were grouped with two starfish species (*A. planci* and *P. miniata*) from the order Valvatida. Expansion and contraction of gene families were evaluated by CAFE v5^63^ with a p-value of 0.05. A total of 1,162 gene families were expanded while 55 were contracted in the deep-sea starfish, *Z*. cf. *ophiactis* (Fig. 4).

## Data Availability

No specific code was used in this study. All commands and pipelines used in the data processing were performed according to manuals and protocols of corresponding bioinformatics software, with parameters described in the Methods section. If no detailed parameters were mentioned for a software, default parameters were used.
